# The Direct Piezoelectric Effect in Deep Eutectic Solvents

**DOI:** 10.1021/jacs.5c21126

**Published:** 2026-02-03

**Authors:** Allison M. Stettler, Sheryl S. Blanchard, Gary A. Baker, G. J. Blanchard

**Affiliations:** † 3078 Michigan State University, Department of Chemistry, East Lansing, Michigan 48824, United States; ‡ University of Missouri − Columbia, Department of Chemistry, Columbia, Missouri 65211, United States

## Abstract

We report the direct piezoelectric response of four deep eutectic solvents (DESs): choline chloride:ethylene glycol (ChCl:EG), choline chloride:glycerol (ChCl:Gly), choline chloride:1,3-propanediol (ChCl:PD), and choline chloride:urea (ChCl:urea). Measurement of current as a function of applied force produces a linear relationship from which the piezoelectric coefficient (*d*_33_) was determined. The piezoelectric effect has previously been observed in room-temperature ionic liquids (RTILs), attributable to a pressure-induced liquid-to-crystalline solid phase transition. The observation of this phenomenon in DESs is unprecedented and underscores its generality. The magnitude of *d*_33_ in these DESs is similar to that for RTILs, suggesting the potential to tune the piezoelectric response through careful selection of the DES constituents and constituent ratios.

## Graphical abstract

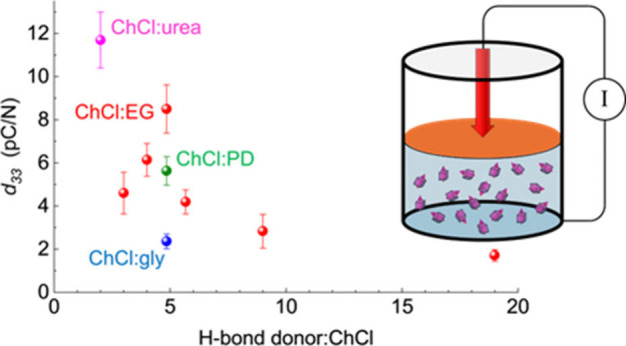

The piezoelectric effect, first discovered in 1880,^2^ is used in multiple applications, from spark sources and inkjet printing to actuators and biosensors. The term “piezoelectric effect” refers to the direct and converse piezoelectric effects, with the direct effect requiring application of force to a material to cause structural distortion resulting in charge separation. This effect requires noncentrosymmetric regular or crystalline structures, e.g., quartz, LiNbO_3_, BaTiO_3_, Pb­[Zr_*x*_Ti_1–*x*_]­O_3_ (0 ≤ *x* ≤ 1) (PZT), certain ceramics, nanomaterials, polymers, and composites.^3–7^ The direct piezoelectric effect has also been reported in some noncrystalline materials, including DNA, viral proteins, and amino acids.^8–13^ Piezoelectric materials will also manifest the converse piezoelectric effect, wherein the application of an electric charge induces mechanical deformation. The converse piezoelectric effect underpins many commercial technologies, including inkjet printing, nanoactuators, and small-amplitude motion-control devices.

The piezoelectric effects are observed in solid-state materials, including selected molecular solids.^14,15^ We recently demonstrated that piezoelectric effects can be observed in room-temperature ionic liquids (RTILs). While the piezoelectric effect is manifested in solids, the RTILs produce the direct piezoelectric effect because they undergo a pressure-induced, reversible liquid to crystalline solid phase transition.^1,16,17^ We report here the first observation of the direct piezoelectric effect in deep eutectic solvents (DESs). This work underscores the generality of this effect and demonstrates for the first time the ability to control the piezoelectric response stoichiometrically. DESs are mixtures of two or more constituents that, upon mixing, display a melting point significantly lower than that of either individual constituent. DESs were first reported in 2003.^18^ Their formation is driven by prominent hydrogen bonding interactions between constituents with contributions from van der Waals, electrostatic, and charge transfer interactions.^19–21^ The term DES encompasses a number of systems with five general archetypes having been identified.^19–21^ The most widely studied is the Type III DES family, composed of a quaternary ammonium cation as the H-bond acceptor and an amide, carboxylic acid, alcohol, sugar, or amino acid as the H-bond donor. Type III DESs are useful because they are biodegradable and display low flammability, toxicity, and volatility compared to common organic solvents.^19,20,22,23^ DES constituents are typically inexpensive, and DESs can be synthesized using simple, solvent-free methods that achieve 100% atom efficiency.^23–25^ Reline, for example, is a 1:2 molar ratio of choline chloride (ChCl) and urea.^18,19^ DESs are effective solvents for biomass processing, redox battery production, metals processing, electroplating, fossil fuel desulfurization, nanochemistry, micellar chemistry, CO_2_ gas capture, and biocatalysis.^19–22,26–31^

Because RTILs exhibit the piezoelectric effect, a key question is, what other materials exhibit pressure-induced liquid-to-crystalline solid phase transitions? The widely studied DES choline chloride:ethylene glycol (ChCl:EG) (1:2) undergoes crystallization at approximately 2.6 GPa,^32^ and pressure-induced phase transitions are well documented for multiple DESs.^33–37^ We report here that several DESs exhibit a direct piezoelectric response under applied pressure, with the magnitude of this response depending on both the identity of the constituents and their molar ratios.

Detailed descriptions of the synthesis of each DES have been reported elsewhere.^38–41^ We briefly recap them here.

## Preparation of ChCl:EG DES

ChCl:EG DESs were prepared using choline chloride (ChCl, Sigma-Aldrich, BioUltra, ≥99.0%) and ethylene glycol (EG, Sigma-Aldrich, ReagentPlus, ≥99%) that were used as received. Compositions of 5 mol % ChCl (1:19 ChCl:EG), 10 mol % ChCl (1:9 ChCl:EG), 15 mol % ChCl (1:5.67 ChCl:EG), 17.1 mol % ChCl (1:4.85 ChCl:EG), 20 mol % ChCl (1:4 ChCl:EG), 25 mol % ChCl (1:3 ChCl:EG), and 33 mol % ChCl (1:2 ChCl:EG) were prepared as follows: Appropriate masses of ChCl and EG were weighed to an accuracy of ± 0.0001 g on an analytical balance (Mettler Toledo LA204E) in a dry 250 mL round-bottom flask followed by rotary evaporation at 80 °C for 30 min with 100 rpm. The clear, colorless, homogeneous fluids were transferred to precleaned 40 mL EPA vials. These vials were equipped with stirbars, capped with PTFE-faced silicone rubber septa, and further dried overnight at 70 °C under a vacuum with continuous stirring.

## Preparation of ChCl:Gly DES

ChCl and glycerol (Gly) were purchased from Aldrich with the highest purity available and used as received. The ChCl:Gly DES was prepared by mixing the two constituents to yield a sample with a mole fraction of 17.1 mol % ChCl (1:4.85) ChCl:Gly. Appropriate masses of ChCl and glycerol were weighed and prepared in the same manner as the ChCl:EG DESs (*vide supra*).

## Preparation of ChCl:PD DES

ChCl (BioUltra, ≥99.0%; catalog no. 26978) and 1,3-propanediol (PD, 98%; catalog no. P50404) were purchased from MilliporeSigma and used as received. The ChCl:PD DES was prepared by mixing the two constituents to yield a sample with a mole fraction of 17.1 mol % ChCl (1:4.85) ChCl:Gly. Appropriate masses of ChCl and glycerol were weighed and prepared in the same manner as the ChCl:EG DESs (*vide supra*).

## Preparation of ChCl:Urea DES

ChCl (BioUltra, ≥99.0%; catalog no. 26978) and urea (BioUltra grade, 99%; catalog no. 51456) were purchased from MilliporeSigma and used as received. The DES was prepared by mixing ChCl and urea at appropriate mass ratios to yield DESs having a ChCl molar percentage of 33.3%, corresponding to a ChCl:urea molar ratio of 1:2. All other preparatory and drying procedures were identical with those used for the ChCl:PD (1:4.85) formulation.

## Ionic Liquid

The ionic liquid used for comparison was *N*-butylpyridinium bis­(trifluoromethyl-sulfonyl)­imide (C_4_Py TFSI), purchased from Sigma-Aldrich and purified prior to use using a procedure reported elsewhere.^42,43^

## Piezoelectric Response Measurement

Measurement of the magnitude of the direct piezoelectric effect was performed using a custom-built instrument designed and constructed in-house.^1^ This instrument consists of a cylinder–piston assembly that houses the DES sample. The cylinder is stainless steel, and the piston is made of Delrin and incorporates a centrally mounted metal electrode. A Buna-N O-ring provides a seal between the piston and cylinder. When force is applied, care must be taken to maintain a seal that permits the passage of air while preventing leakage of the DES sample. The apparatus operates as a second-class lever system, enabling the controlled application of force to the sample. Current transients generated during the application and release of force are measured as a function of applied force using an electrometer (Keithley 6517B). For this instrument, the voltage burden during closed circuit current measurements was determined to be 77 μV, corresponding to a leakage current of approximately 77 fA, at least 6 orders of magnitude lower than the measured currents reported here. Electrometer control and data acquisition are performed by using a LabVIEW virtual instrument (VI) developed in-house. The applied force was measured with a calibrated digital force gauge (Nidec model FG-3009).

Given the operative mechanism underlying the direct piezoelectric response of RTILs, there is no reason to believe that this behavior is unique or limited to ionic liquids. In principle, any material that undergoes pressure-induced liquid-to-crystalline solid phase transitions could exhibit a direct piezoelectric response. On this basis, we investigated DESs as potential piezo-active liquids. The 1:4.85 ChCl:EG system was found to exhibit a clear, measurable direct piezoelectric current–force response (Figure 1). To verify that this behavior is not an electronic artifact, Figure 2 shows the corresponding potential–force response. The observation of both the potential–force (energy storage) and current–force (energy release) behavior confirms the piezoelectric nature of the response.^1^ We next examined how the magnitude of the piezoelectric coefficient (*d*_33_) varies with the composition of DES systems.

**Figure 1:**
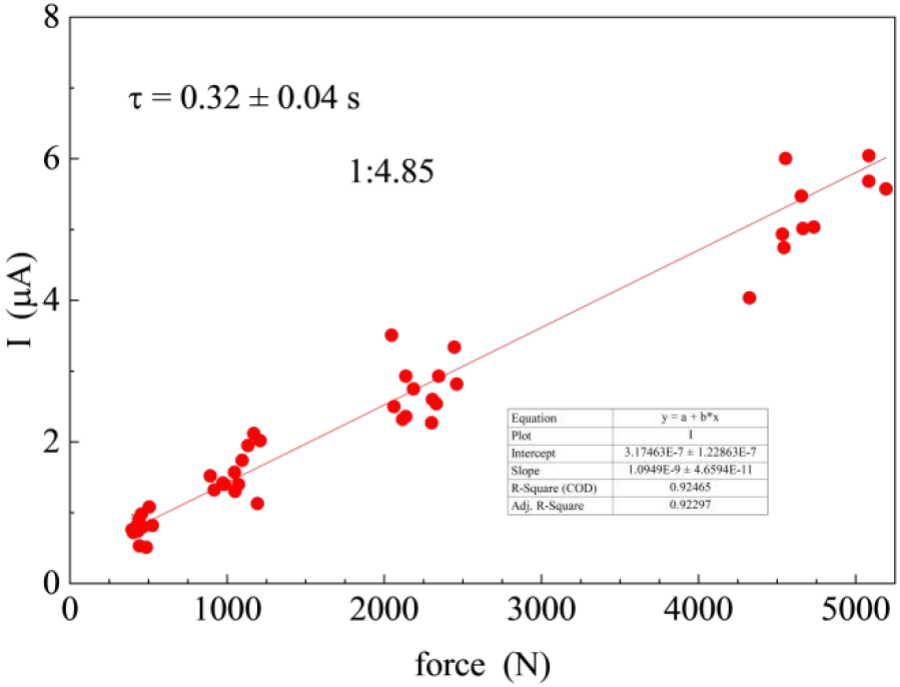
Current vs applied force response for 1:4.85 ChCl:EG DES.

**Figure 2:**
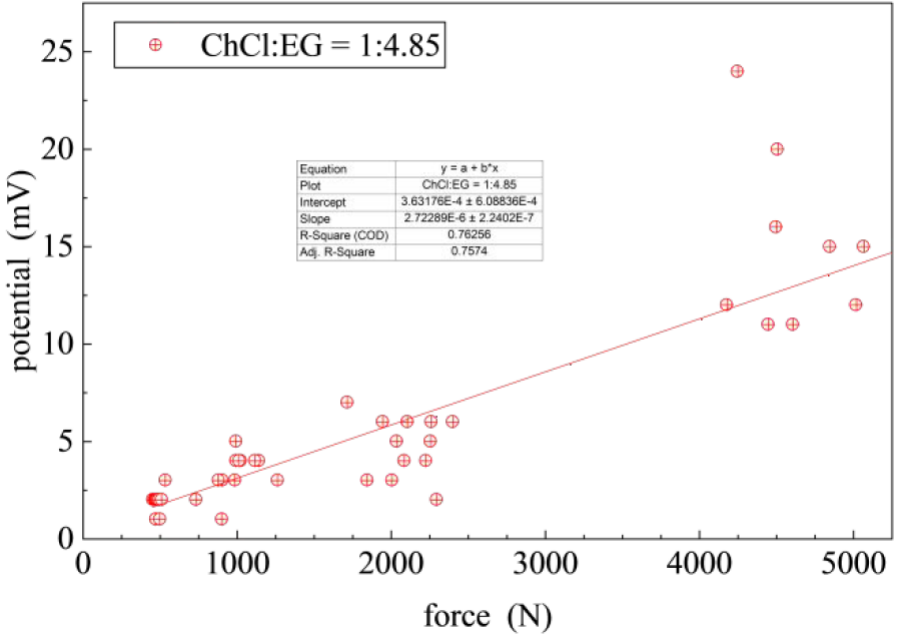
Potential vs applied force response for 1:4.85 ChCl:EG DES.

The first consideration concerns the relationship between the magnitude of the piezoelectric response and the composition of the DES system. We note that the term “deep eutectic solvent” currently lacks a precise, universally accepted definition and generally refers to binary or more complex mixtures that exhibit a melting-point reduction relative to their individual components. In practice, these mixtures typically exhibit a negative deviation from ideality, driven by favorable enthalpic interactions (e.g., strong hydrogen bonding) rather than purely entropic effects. At least one component is often a solid under ambient conditions, making the observed liquification upon mixing physically meaningful, although many researchers now extend the definition to include systems composed entirely of liquid constituents. Recent work from the Panzer group has proposed a quantitative threshold based on the molar excess Gibbs energy to distinguish true DESs from conventional eutectic mixtures; however, this criterion has not yet seen broad adoption.^44^ Although the ratio of DES constituents can vary and only a single composition exhibits the minimum melting point, the term “DES” is generally applied to all constituent ratios. The “true” eutectic composition for ChCl:EG has been determined to be ∼17.1% ChCl, a molar ratio of 1:4.85 (ChCl:EG).^45^ Interestingly, our recent work has revealed a distinctive structural relationship between the H-bond donor and acceptor components at this composition for EG-, Gly-, and PD-based systems.^38–40^ Owing to the unique behavior exhibited by these DESs at this ratio, it was selected for initial evaluation of the piezoelectric response, and ChCl:EG was chosen due to its lower viscosity relative to ChCl:Gly and ChCl:PD. For the 1:4.85 ChCl:EG system, the piezoelectric coefficient *d*_33_ was determined from the slope of the current–force response (*dq/dt*/force) (Figure 1) and the duration of the current transients (*dt*) to be 8.5 ± 1.1 pC/N (((*dq*/*dt*)×*dt*)/force), as described previously.^1^ By comparison, the *d*_33_ value for the RTIL C_4_Py TFSI is 18.1 ± 1.3 pC/N (Figure S9).^1^ The DESs investigated here can generate piezoelectric responses similar in magnitude to those observed with RTILs. The recovered slopes and pulse widths (Table 1) appear to yield higher *d*_33_ values, but these data were acquired with higher gain used in the current measurement than was used in our earlier report.^1^ We have normalized the *d*_33_ values to be the same as that reported previously for C_4_Py TFSI, which we believe to be more accurate.

**Table 1:** *d*_33_ Values for DESs Determined from Current–Force Data and Current Transient Widths as Functions of DES Identity and Constituent Ratio

DES	Current Pulse Width (s)	Experimental Slope (nA/N)	*d*_33_ (pC/N)^a^
ChCl:EG 1:3.00	0.30 ± 0.05	0.63 ± 0.08	4.6 ± 1.0
ChCl:EG 1:4.00	0.29 ± 0.03	0.87 ± 0.06	6.1 ± 0.8
ChCl:EG 1:4.85	0.32 ± 0.04	1.09 ± 0.05	8.5 ± 1.1
ChCl:EG 1:5.67	0.33 ± 0.03	0.52 ± 0.05	4.2 ± 0.6
ChCl:EG 1:9.00	0.21 ± 0.05	0.55 ± 0.08	2.8 ± 0.8
ChCl:EG 1:19.00	0.25 ± 0.03	0.28 ± 0.03	1.7 ± 0.3
ChCl:Gly 1:4.85	0.44 ± 0.05	0.22 ± 0.02	2.4 ± 0.3
ChCl:PD 1:4.85	0.42 ± 0.03	0.55 ± 0.05	5.6 ± 0.7
ChCl:urea 1:2.0	0.22 ± 0.03	0.15 ± 0.02	11.7 ± 1.3
C_4_Py TFSI	0.32 ± 0.05	2.31 ± 0.12	18.1 ± 1.3

a The values of *d*_33_ are normalized to the value of *d*_33_ for C_4_Py TFSI, which was determined previously to be 18.1 ± 1.3 pC/N.^1^

The piezoelectric coefficients of the ChCl:polyol DES systems vary with both the constituent molar ratio (Figures S1−S6) and the identity of the polyol HBD component (Figures S7 and S8). These results are summarized in Table 1 and Figure 3. Several notable trends emerge from these data. First, the nominal “true” eutectic composition for ChCl:EG corresponds to the highest measured *d*_33_ value, with *d*_33_ decreasing as the composition deviates from this ratio. This could be the result of either constituent stoichiometry- or composition-dependent changes in the pressure-dependent DES phase diagram. Resolution of this question is under examination. Second, a measurable direct piezoelectric response is also evident for both ChCl:Gly and ChCl:PD systems, underscoring the generality of the phenomenon and suggesting the importance of constituent structures in optimizing the piezoelectric response.

**Figure 3:**
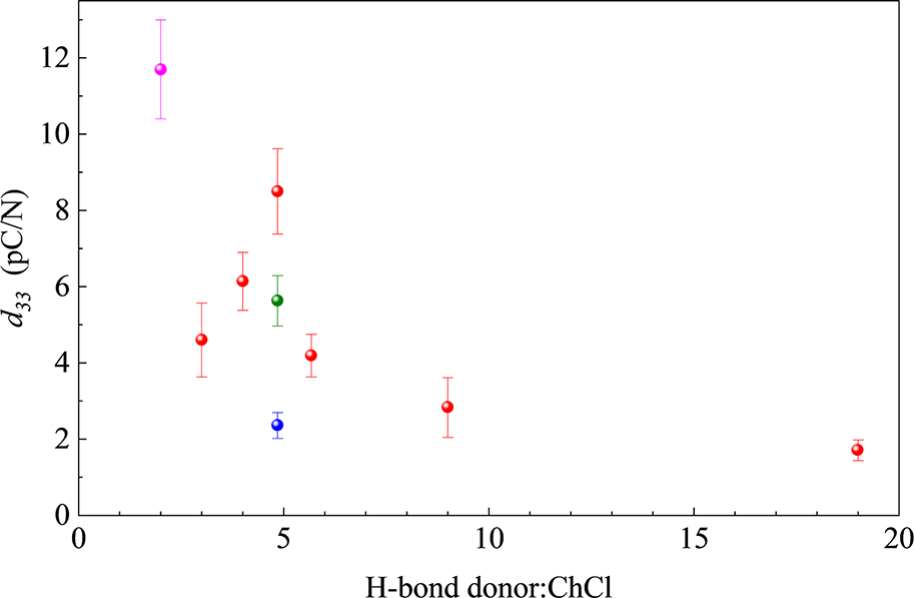
*d*_33_ values determined from experimental current–force (*I* vs *F*) data as a function of H-bond donor:ChCl molar ratio and H-bond donor identity. ChCl:EG systems in red, ChCl:PD in green, ChCl:gly in blue, and ChCl:urea in magenta.

We also measured the direct piezoelectric response of the DES reline (ChCl:urea 1:2) given its status as the most extensively studied DES. The *d*_33_ value for reline is 11.7 ± 1.3 pC/N, similar to that of the other DESs examined. It is important to note that reline is known to exist as a metastable liquid^46^ and will solidify at room temperature over an extended period of time. The initial piezoelectric studies with reline showed that it was in the liquid phase before and after the application of force, but subsequent measurements of the same batch of reline, after having gone through room temperature liquid-to-solid and remelting sequences, showed a propensity to produce solid material upon exposure to force in the piezoelectric apparatus. This behavior for reline stands in contrast to that of the ChCl:polyol systems we have reported here. Further investigation is required to understand whether there are long-term organizational effects associated with either the application of pressure or temperature cycling in this system.

We found that DESs exhibit a direct piezoelectric response upon application of pressure. The largest *d*_33_ value was observed for the 1:4.85 ChCl:polyol compositions with ChCl:EG displaying the highest response (8.5 pC/N), followed by ChCl:PD (5.6 pC/N) and ChCl:Gly (2.4 pC/N). The nonpolyol reline mixture (1:2 ChCl:urea) produced a *d*_33_ response of 11.7 pC/N. The first liquid-phase systems reported to exhibit a direct piezoelectric response were room-temperature ionic liquids, where the operative mechanism was attributed to pressure-induced liquid-to-crystalline-solid phase transitions.^16,17^ We propose that this mechanism is more general in nature and extends beyond ionic liquids, as demonstrated by the results presented here for a series of conventional hydrophilic DESs. These findings parallel earlier observations of dynamic heterogeneity in DESs,^38–40^ suggesting a correlation between piezoelectric response and proximity to the eutectic composition. These results open new opportunities for designing tailored materials whose piezoelectric behavior can be tuned through the molecular structure and pressure-induced phase-transition characteristics.

## Supplementary Material



## Notes

Current transient amplitude vs applied force plots for ChCl:EG (1:3.0, 1:4.0, 1:4.85, 1:5.67, 1:9.0, and 1:19 ratios), ChCl:Gly (1:4.85), ChCl:PD (1:4.85), ChCl:urea (1:2), and C_4_Py TFSI (PDF)

## Abbreviations

RTIL: room temperature ionic liquid;
DES: deep eutectic solvent
ChCl: choline chloride
EG: ethylene glycol
Gly: glycerol
PD: 1,3-propanediol
