# Supplementary material for: The Direct Piezoelectric Effect in Deep Eutectic Solvents
Source: J Am Chem Soc. 2026 Feb 3;148(6):5900–4. doi: 10.1021/jacs.5c21126 (PMC12921850; doi:10.1021/jacs.5c21126)
Supplement: Supplementary file 1 [file ja5c21126_si_001.pdf]

Supporting information

The Direct Piezoelectric Effect in Deep Eutectic Solvents

Allison M. Stettler,[1] Sheryl S. Blanchard,[1] Gary A. Baker[2] and G. J. Blanchard[1,*]

[1] Michigan State University, Department of Chemistry, East Lansing, MI 48824-1322 USA

[2] University of Missouri-Columbia, Department of Chemistry, Columbia, MO 65211 USA

Figure S1. Current vs. force graph for 1:3.0 ChCl:EG.

Figure S2. Current vs. force graph for 1:4.0 ChCl:EG.

Figure S3. Current vs. force graph for 1:4.85 ChCl:EG.

Figure S4. Current vs. force graph for 1:5.67 ChCl:EG.

Figure S5. Current vs. force graph for 1:9.0 ChCl:EG.

Figure S6. Current vs. force graph for 1:19 ChCl:EG.

Figure S7. Current vs. force graph for 1:4.85 ChCl:Gly.

Figure S8. Current vs. force graph for 1:4.85 ChCl:PD.

Figure S9. Current vs. force graph for C_4Py TFSI.

Figure S10. Current vs. force graph for 1:2 ChCl:urea.

Figure S11. Current vs. time data for *n*-heptane

Figure S12. Current vs. time data for ethylene glycol.

[*] Corresponding author. Email: blanchard@chemistry.msu.edu, Tel: +1 517 353 1105.

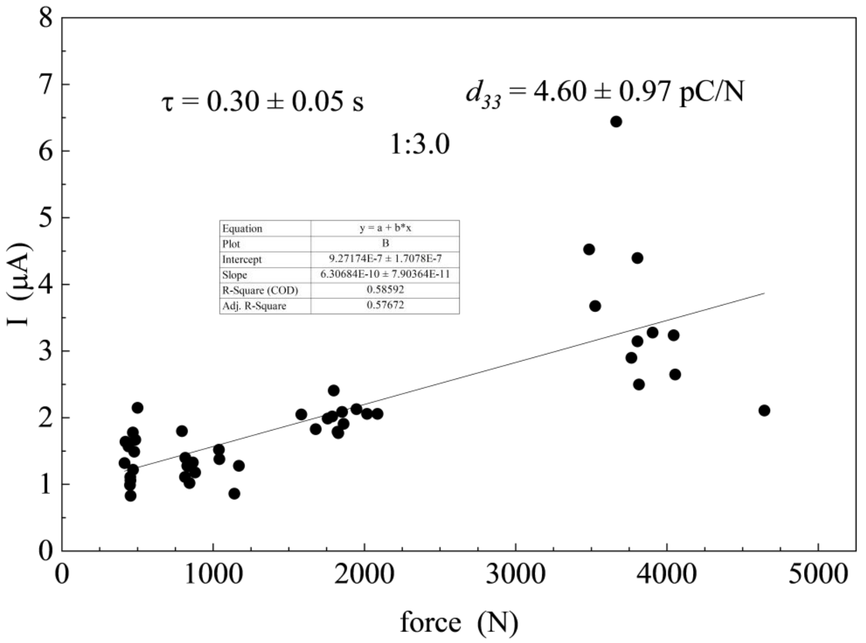

Figure S1. Current vs. force for 1:3 ChCl:EG.

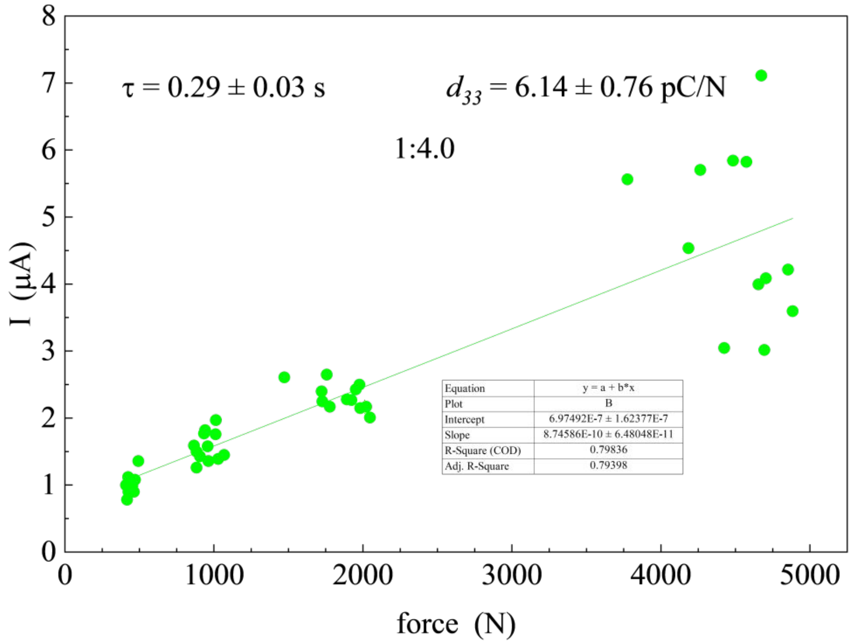

Figure S2. Current vs. force for 1:4 ChCl:EG.

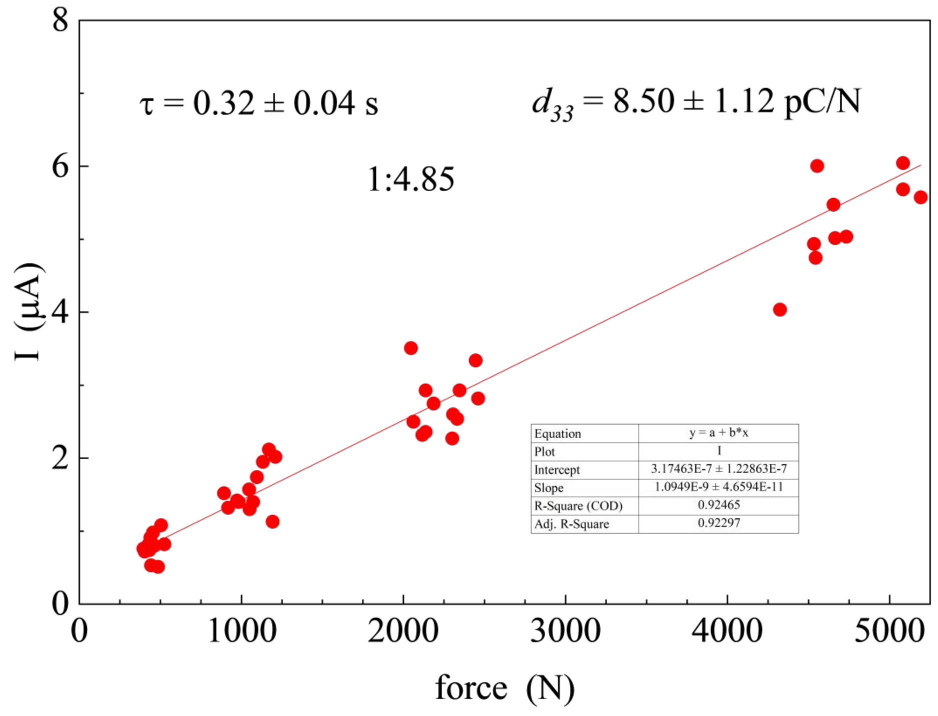

Figure S3. Current vs. force for 1:4.85 ChCl:EG.

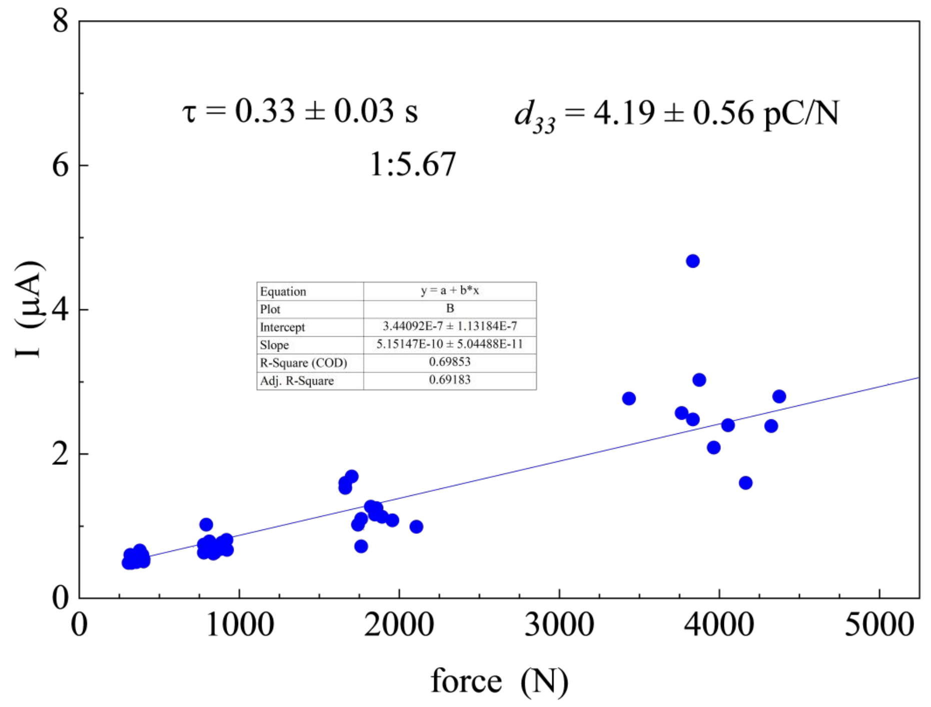

Figure S4. Current vs. force for 1:5.67 ChCl:EG.

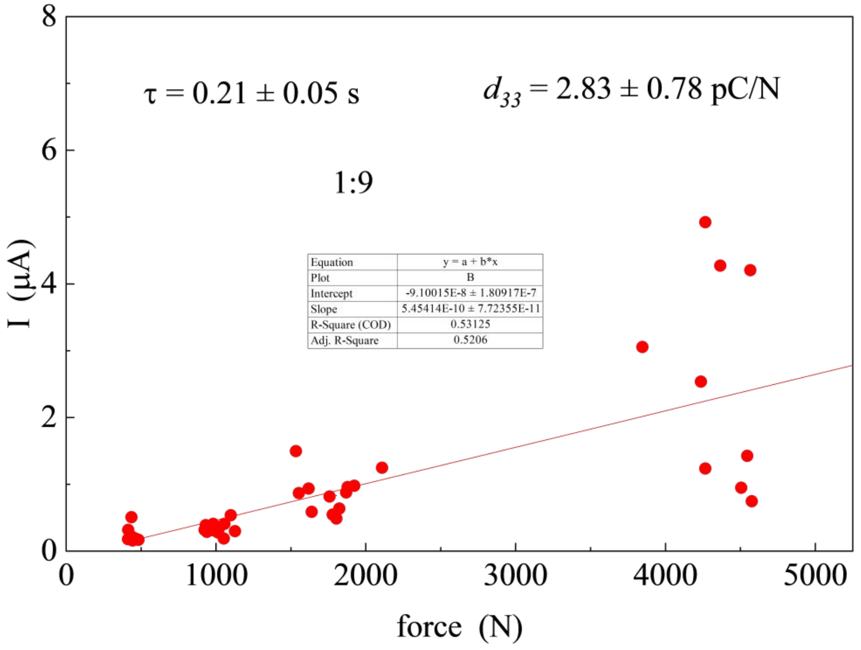

Figure S5. Current vs. force for 1:9.0 ChCl:EG.

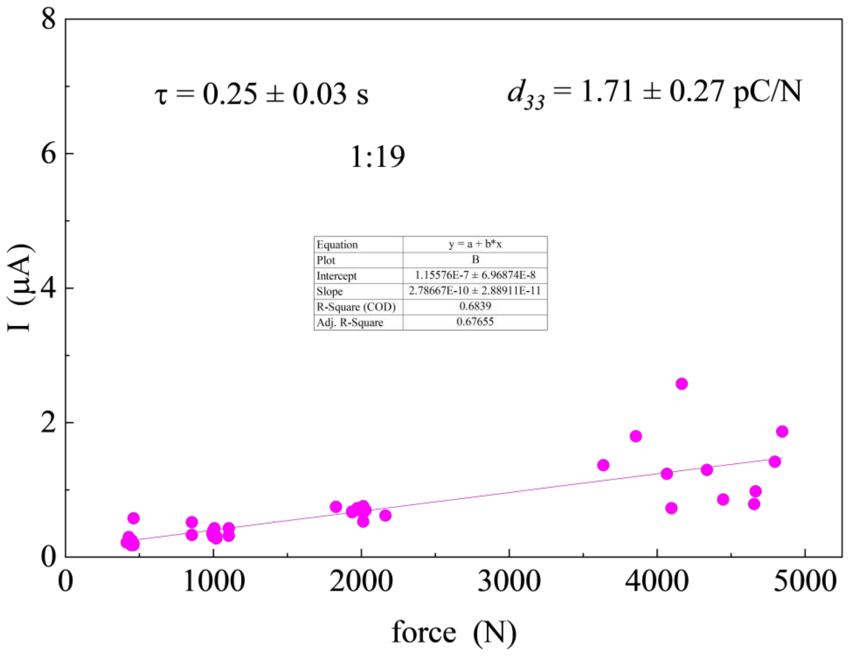

Figure S6. Current vs. force for 1:19 ChCl:EG.

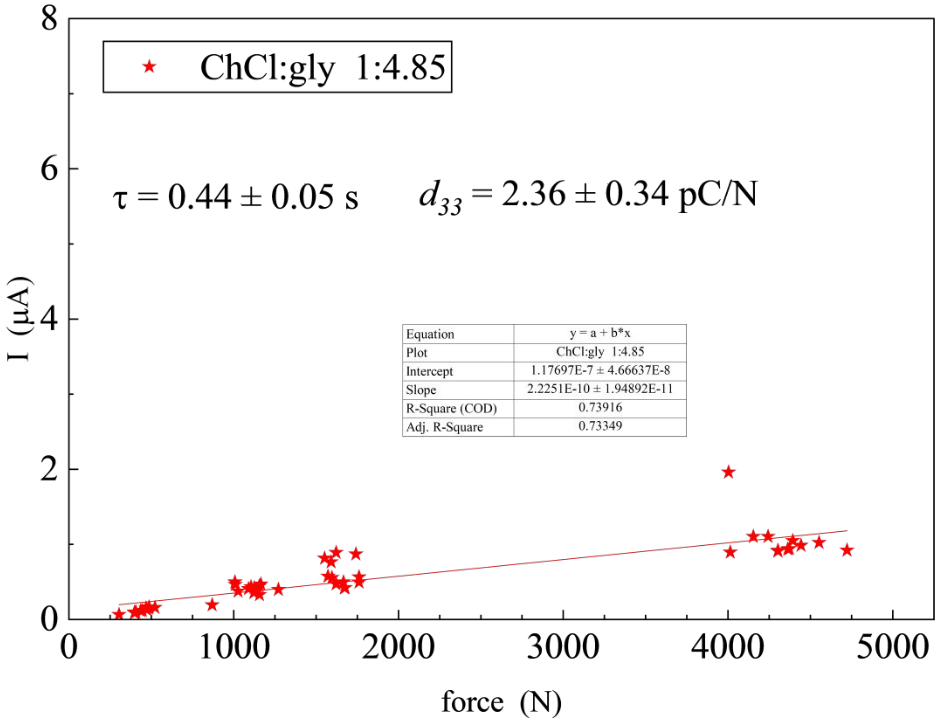

Figure S7. Current vs. force for 1:4.85 ChCl:Glycerol.

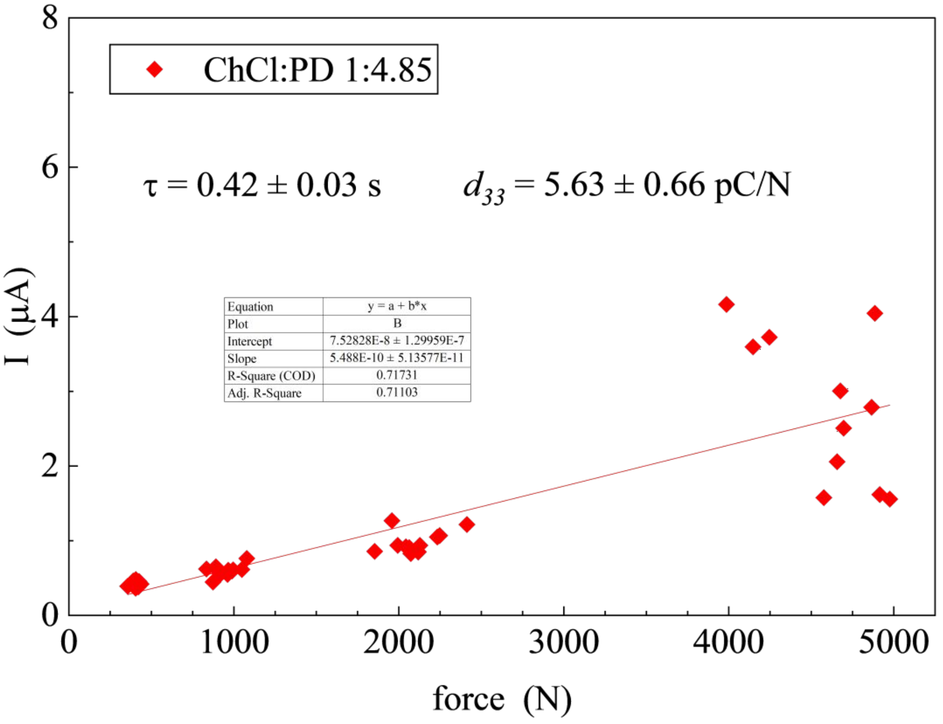

Figure S8. Current vs. force for 1:4.85 ChCl:1,3-propandiol.

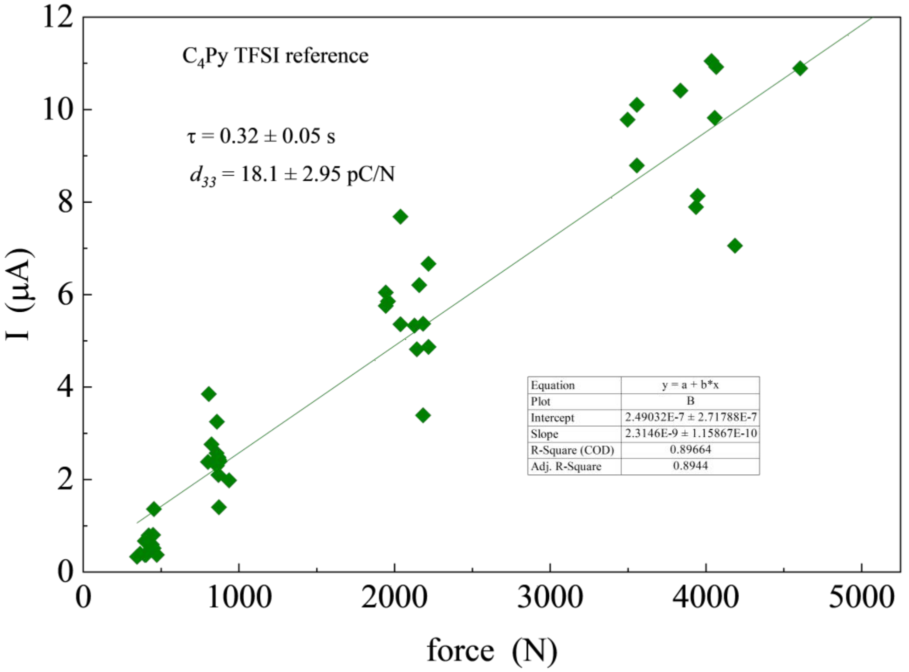

Figure S9. Current vs. force for the RTIL C4Py TFSI.

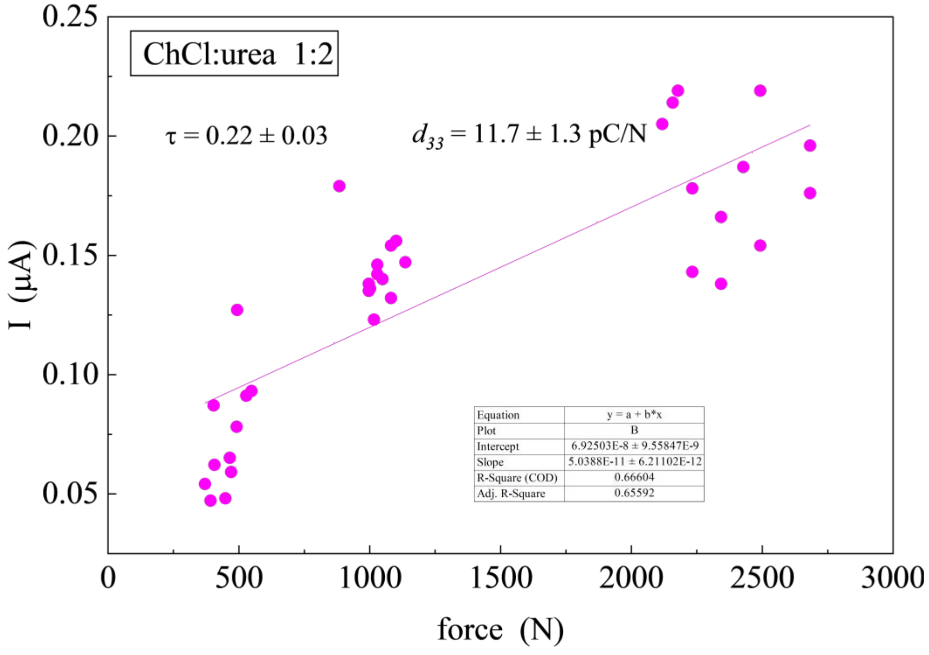

Figure S10. Current vs. force for 1:2 ChCl:urea.

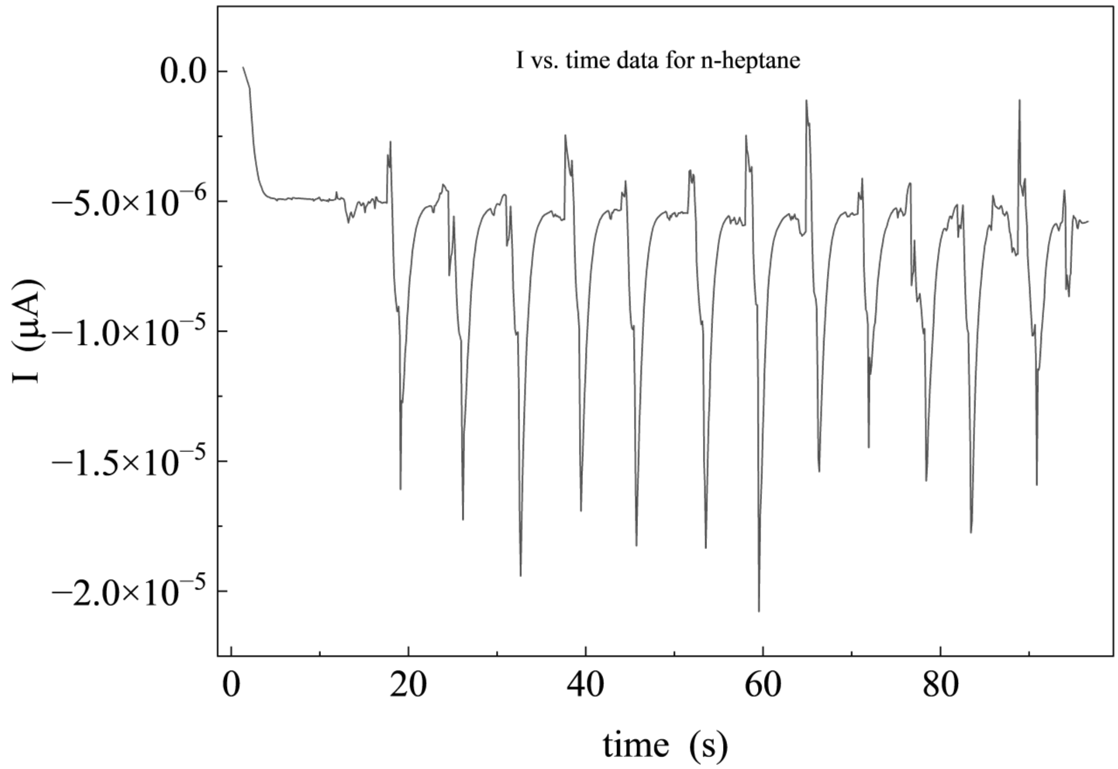

Figure S11. Current vs. time data for *n*-heptane. The signal is associated with the conductivity of the sample, which is limited by the presence of adventitious water in the system. The magnitude of the current signal is independent of the force applied, *i.e.*, $d_{33} = 0$.

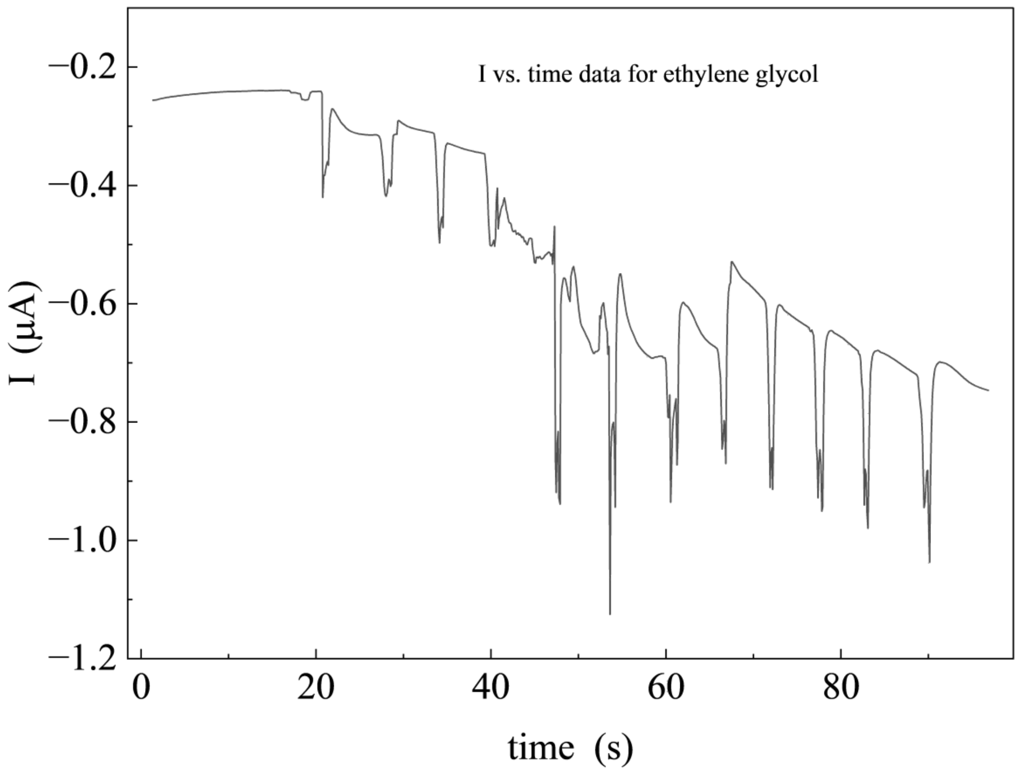

Figure S12. Current vs. time data for ethylene glycol. The signal is associated with the conductivity of ethylene glycol and any water present in the system. The magnitude of the current signal is independent of the force applied, *i.e.*, $d_{33} = 0$.
